# Supplementary material for: An enigmatic decoupling between heat stress and coral bleaching on the Great Barrier Reef
Source: PeerJ. 2019 Aug 12;7:e7473. doi: 10.7717/peerj.7473 (PMC6694787; doi:10.7717/peerj.7473)
Supplement: Supplemental Information 1 [file peerj-07-7473-s001.pdf]

Supplement

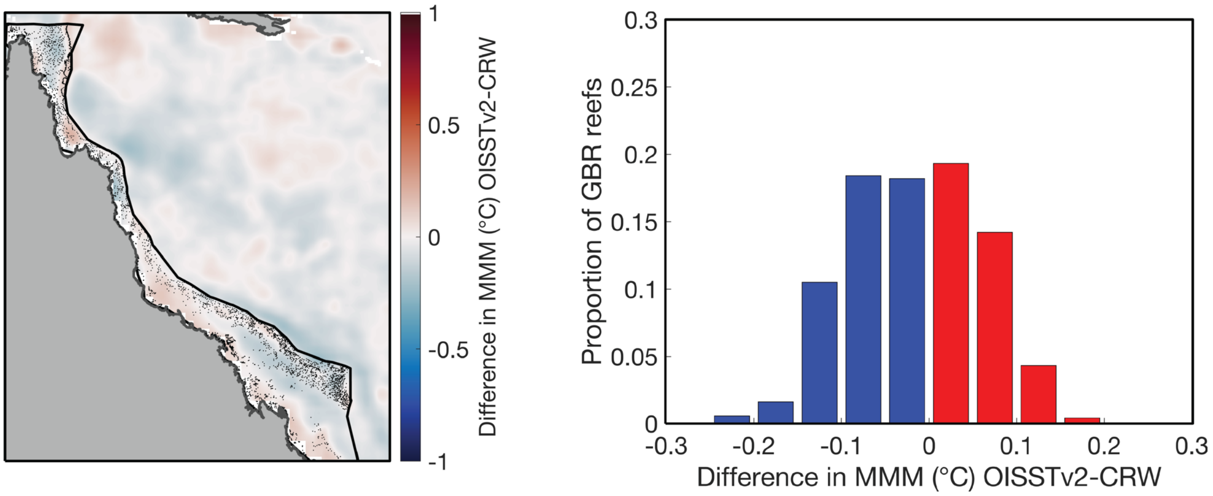

Figure S1. Comparison of MMM climatologies between OI-SSTv2 and CRW. Both climatologies were calculated for the years 1985-2012. Left: map of MMM differences, where red (blue) indicates the OI-SSTv2 climatology is warmer (cooler) than the CRW climatology, which would cause the OI-SSTv2 DHWs calculated with our approach to be lower (higher) than if we used the climatology internal to each product. Right: histogram of MMM differences across the reefs of the GBR. Since there are more reefs with negative differences, the OI-SSTv2 DHWs calculated without our approach using the CRW climatology are slightly higher, on average, than they would be if we used the climatology calculated from OI-SSTv2 itself.

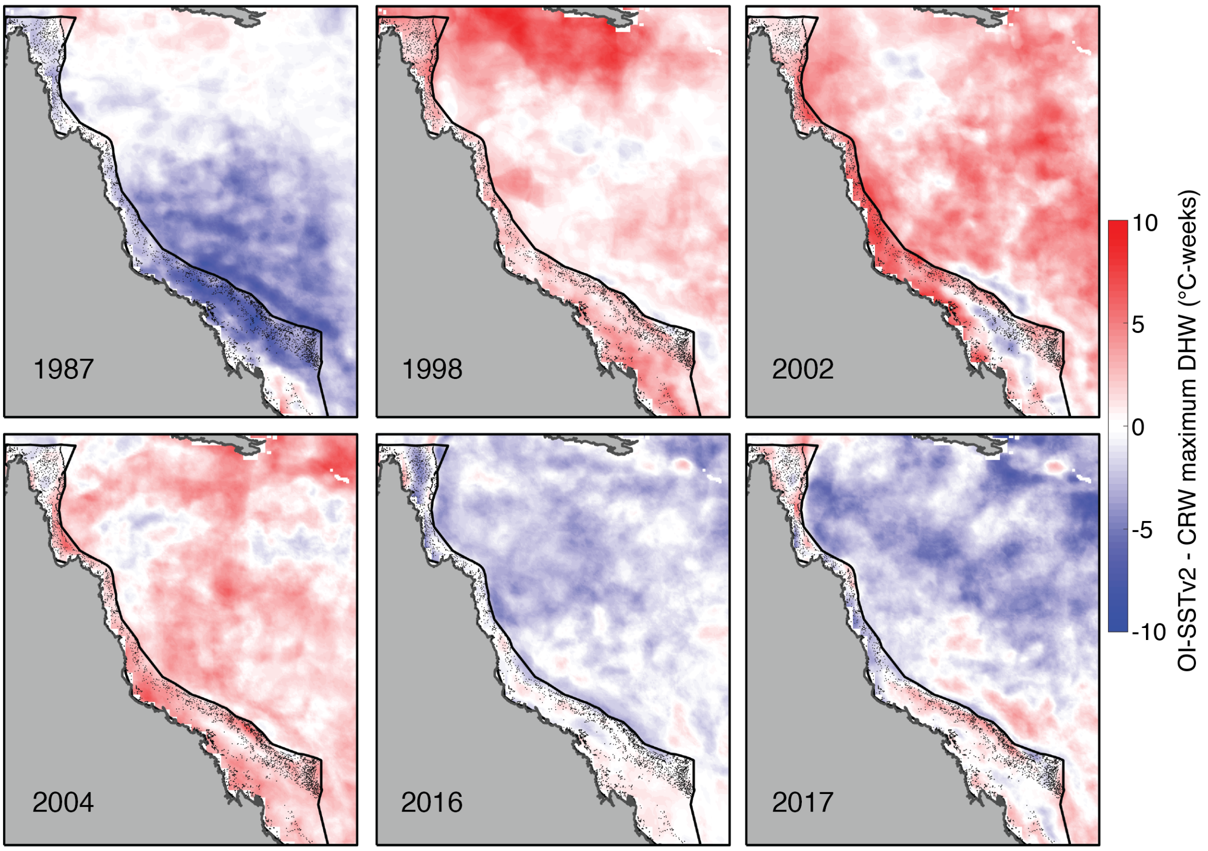

Figure S2. Maps of the differences in maximum DHW between OI-SSTv2 and CRW for 2004 and bleaching years, including the moderate bleaching event in 1987. As described in the main text, DHWs were calculated from each product using a single climatology (the one provided by CRW CoralTemps). The difference in DHW between the two SST products varies dramatically across years and spatially within each year, indicating that these differences are due to differences in the SST variability of each product, not the choice of climatology.

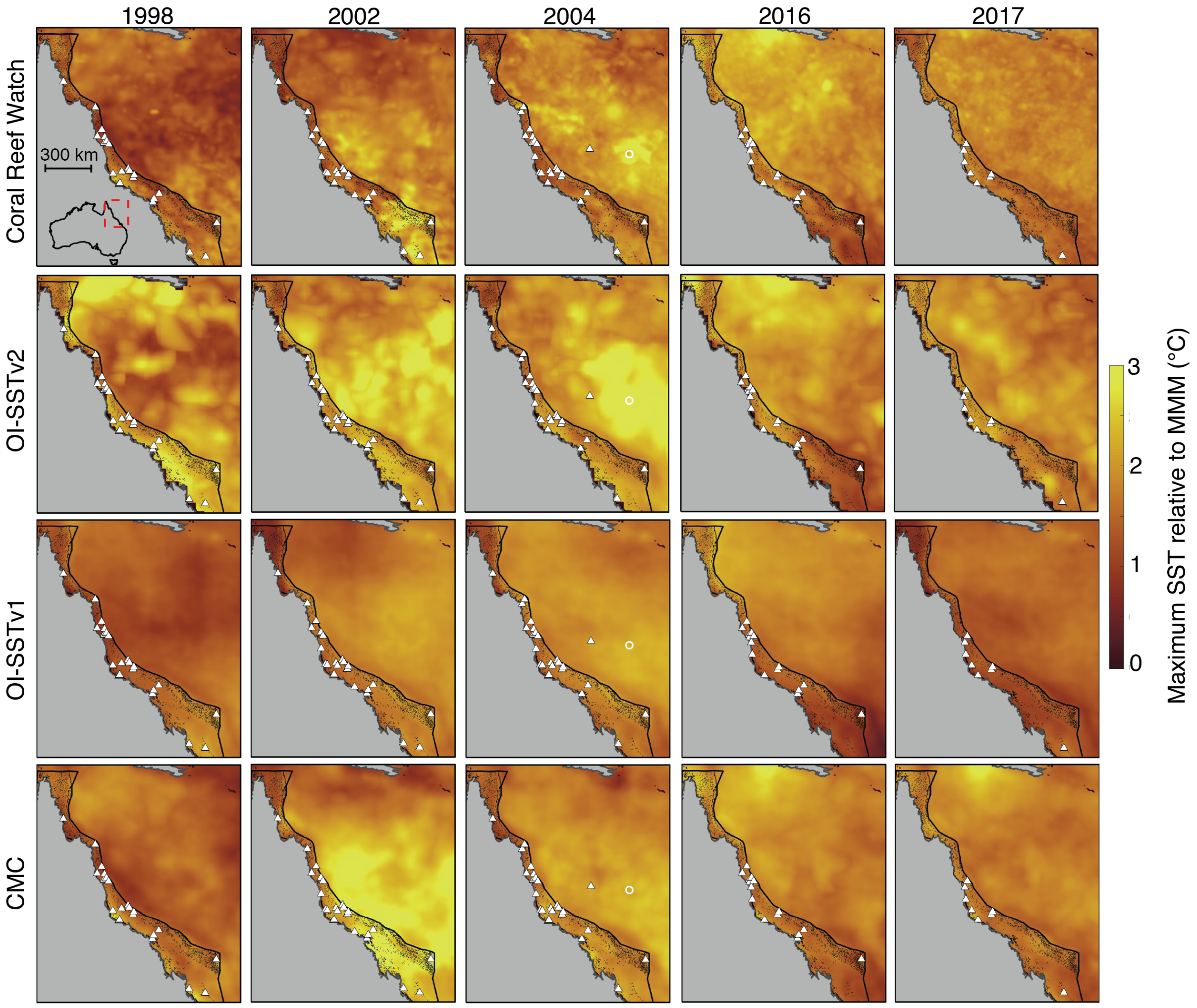

Figure S3. Maximum SST anomalies on the GBR during austral summers of 2004 and coral bleaching years. Rows correspond to different satellite-SST products and columns show the key years assessed in this study. Colors represent the maximum SST relative to the climatological MMM per 5-km pixel during each year. White triangles indicate the locations of *in situ* temperature loggers used each year in the validation of satellite-derived SST. Black dots represent the reefs of the GBR. The white circle in 2004 maps indicates Lihou Reef, where 65% bleaching was observed.

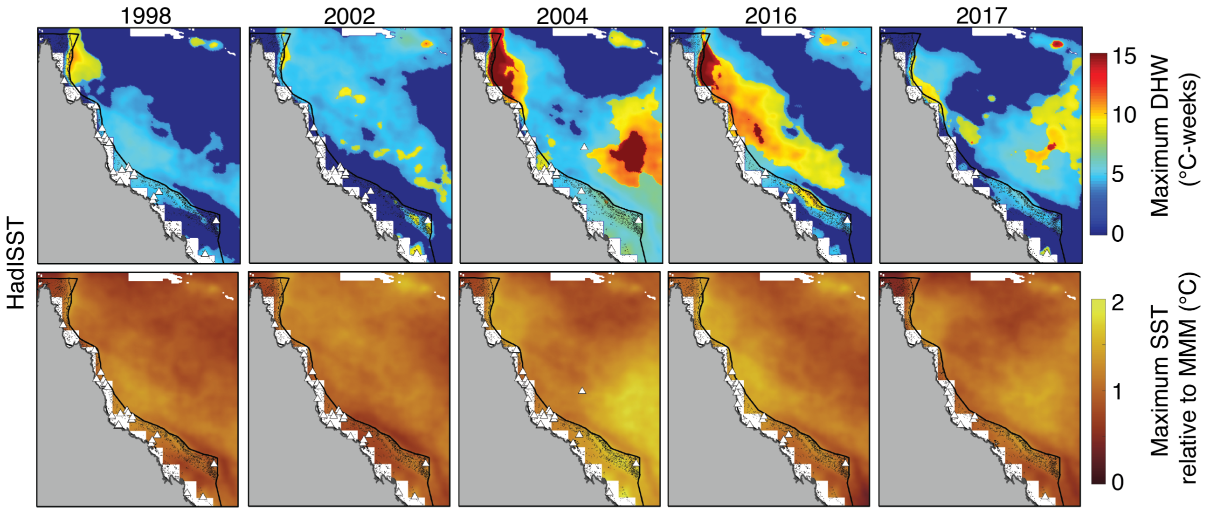

Figure S4. Maximum DHW (top row) and SST anomalies (bottom row) derived from HadISST on the GBR during austral summers of 2004 and coral bleaching years. White triangles indicate the locations of *in situ* temperature loggers used each year in the validation of satellite-derived SST. Black dots represent the reefs of the GBR.

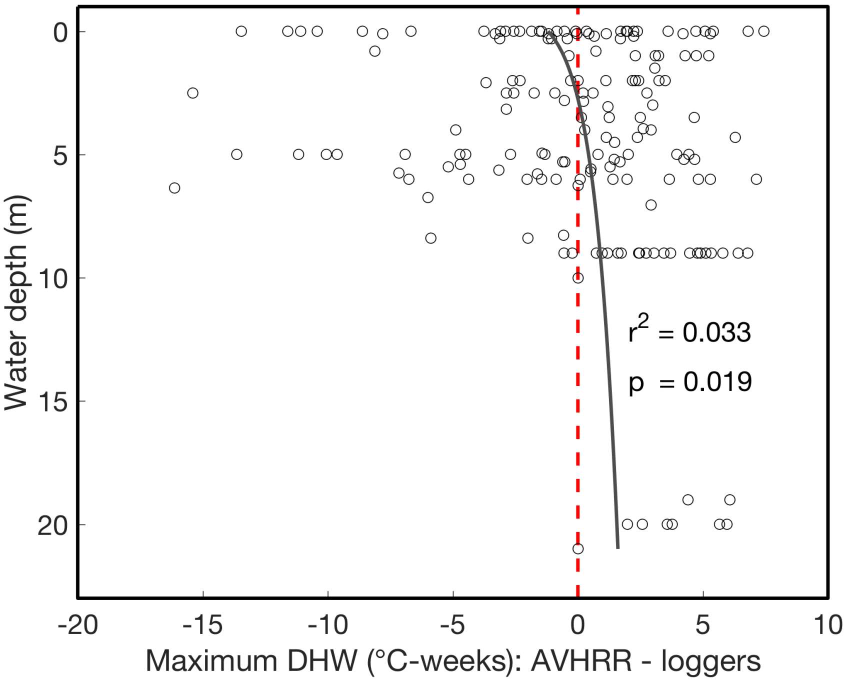

Figure S5. Difference between maximum degree heating weeks (DHW) recorded by OI-SSTv2 and loggers during 2004 and bleaching years (each point is a separate logger in each year).

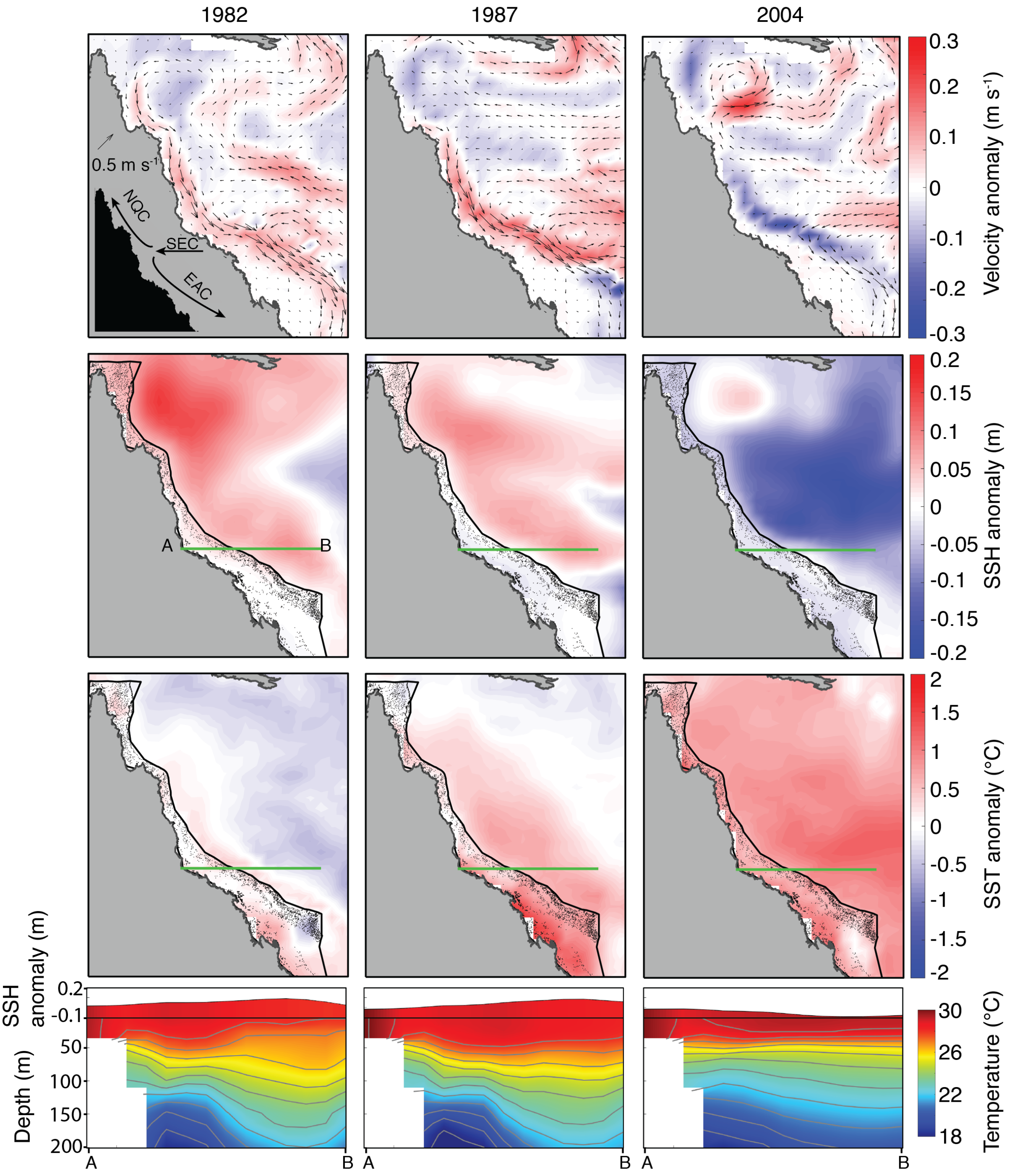

Figure S6. Same as Fig. 9 in the main text, except comparing 2004 to the moderate bleaching events in 1982 and 1987, rather than the severe bleaching events in 1998 and 2002.

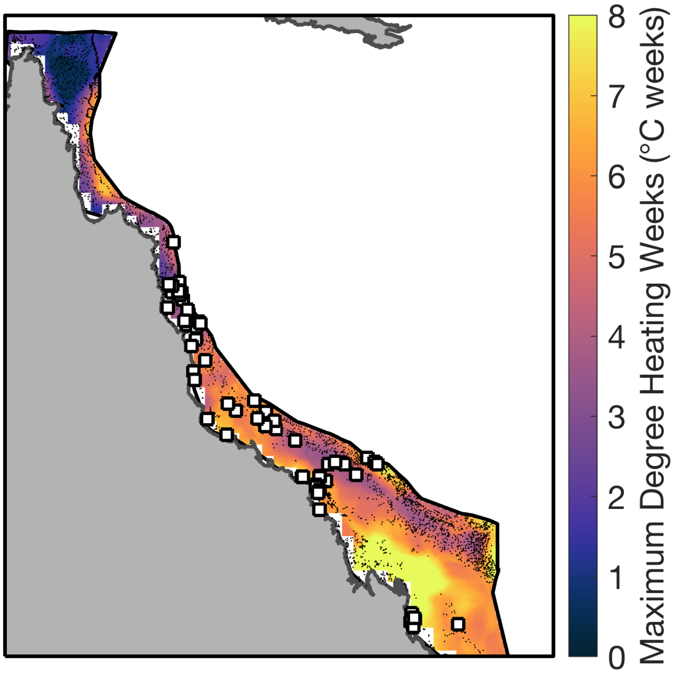

Figure S7. Locations of underwater surveys conducted during January-May 2004 (white squares). The background colors indicate maximum DHW during 2004 based on OI-SSTv2.

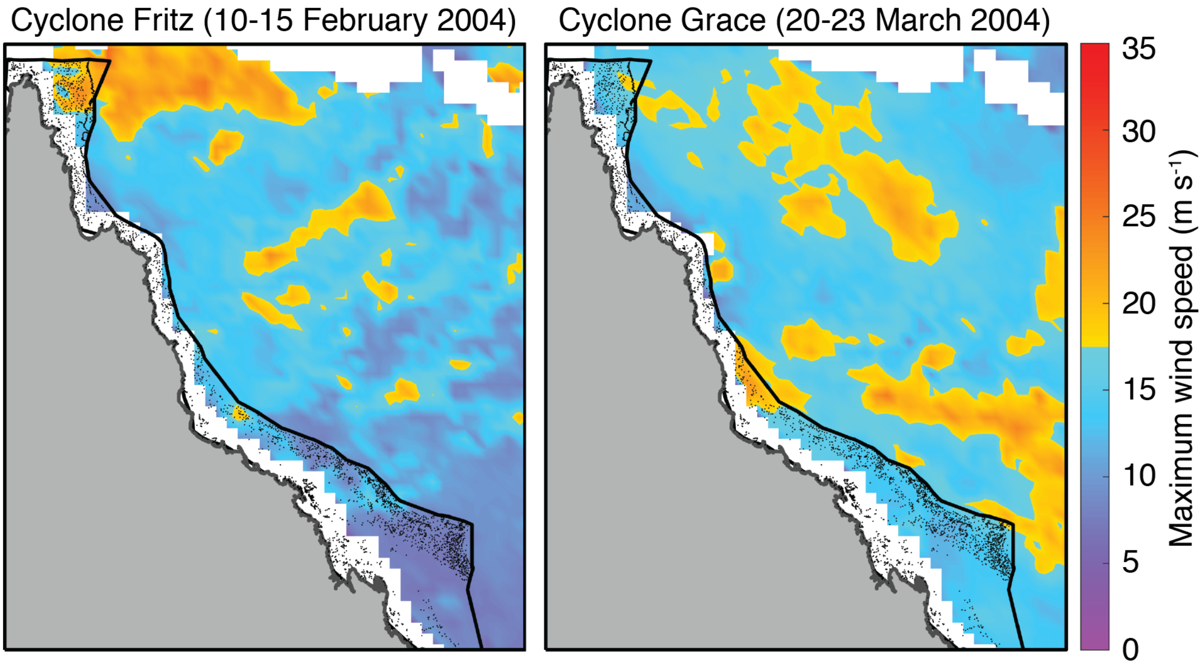

Figure S8. Maximum sustained (12-hr) wind speeds (from QuikSCAT) during Cyclone Fritz (10-15 February 2004) and Cyclone Grace (20-23 March 2004). Orange-red colors indicate cyclone-intensity wind speeds (>34 knots, or 17.5 m s^{-1}).

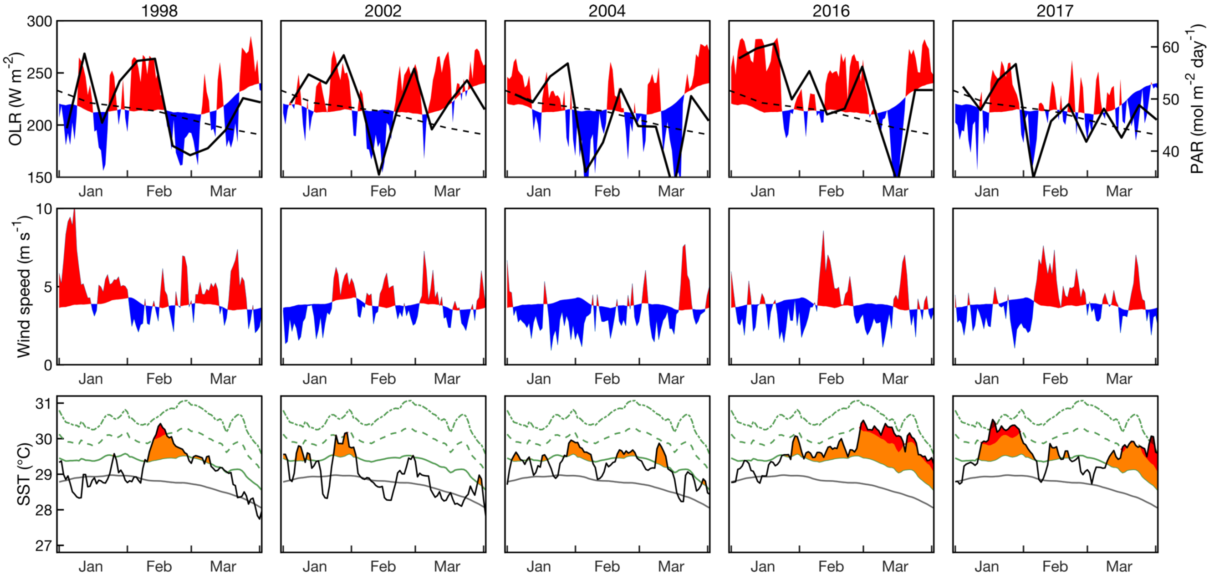

Figure S9. Same as Figure 6 in the main text, but for the northern GBR.

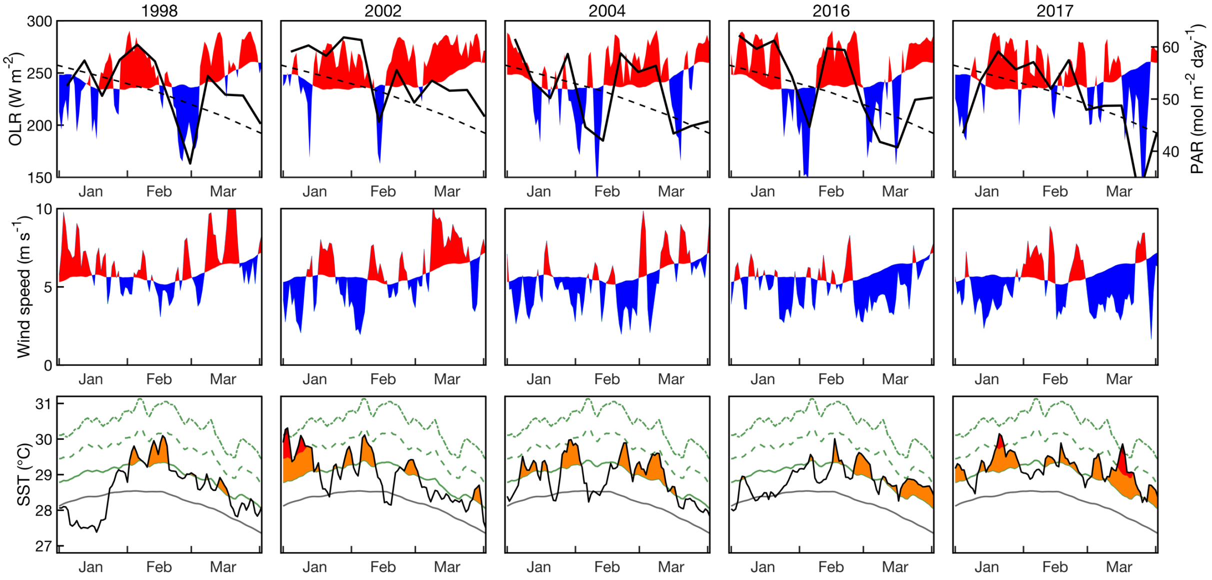

Figure S10. Same as Figure 6 in the main text, but for the central GBR.

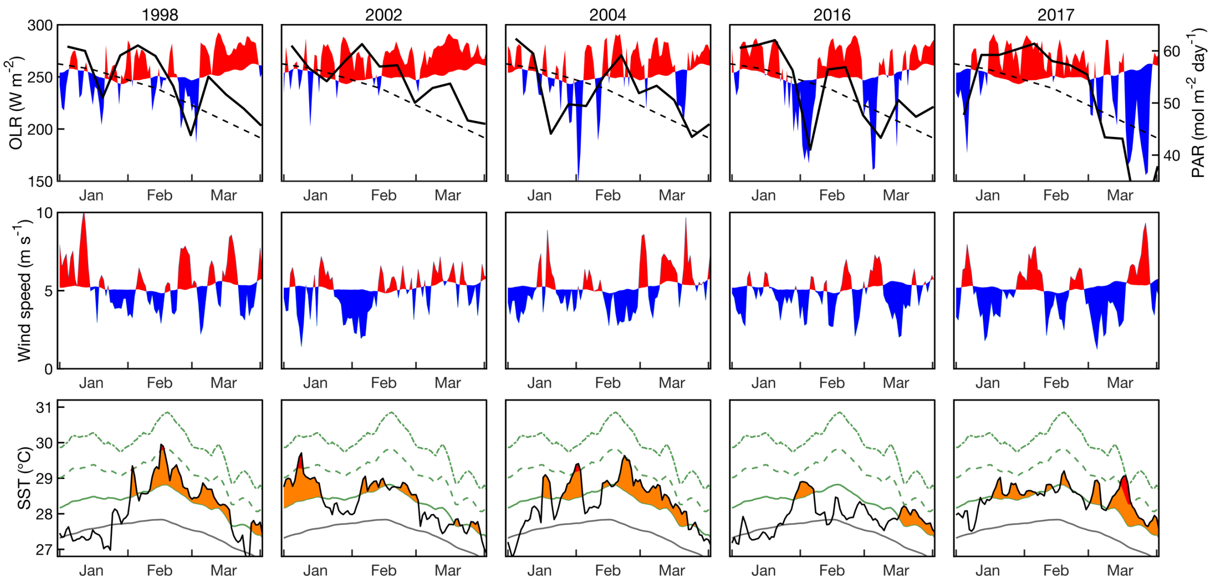

Figure S11. Same as Figure 6 in the main text, but for the southern GBR.
